# The southernmost foci of *Dermacentor reticulatus* in Italy and associated *Babesia canis* infection in dogs

**DOI:** 10.1186/s13071-016-1502-9

**Published:** 2016-04-18

**Authors:** Emanuela Olivieri, Sergio A. Zanzani, Maria S. Latrofa, Riccardo P. Lia, Filipe Dantas-Torres, Domenico Otranto, Maria T. Manfredi

**Affiliations:** 1 Department of Veterinary Medicine, University of Perugia, 06126 Perugia, Italy; 2 Department of Veterinary Medicine, University of Milan, 20133 Milan, Italy; 3 Department of Veterinary Medicine, University of Bari, 70010 Valenzano, Bari Italy; 4 Department of Immunology, Aggeu Magalhães Research Centre Oswaldo Cruz Foundation (Fiocruz), 50740465 Recife, Pernambuco Brazil

**Keywords:** *Dermacentor reticulatus*, Tick, *Babesia canis*, Protozoa, Dog, Italy

## Abstract

**Background:**

Two clustered clinical cases of canine babesiosis were diagnosed by veterinary practitioners in two areas of northeastern Italy close to natural parks. This study aimed to determine the seroprevalence of babesial infection in dogs, the etiological agents that cause canine babesiosis and the potential tick vector for the involved *Babesia* spp.

**Methods:**

The study area was represented by two parks in northeastern Italy: Groane Regional Park (Site A) and the Ticino Valley Lombard Park (Site B). From March to May 2015 ticks were collected from the vegetation in three transects in each site. In the same period, blood samples were collected from 80 dogs randomly chosen from veterinary clinics and kennel located in the two areas. Morphological identification of the ticks was performed and six specimens were molecularly characterised by the amplification and sequencing of partial mitochondrial 12S rRNA, 16S rRNA and *cox*1 genes. For phylogenetic analyses, sequences herein obtained for all genes and those available from GenBank for other *Dermacentor* spp. were included. Dog serum samples were analysed with a commercial indirect fluorescent antibody test to detect the presence of IgG antibodies against *Babesia canis*. Ticks and blood samples were tested by PCR amplification using primers targeting 18S rRNA gene of *Babesia* spp.

**Results:**

Ticks collected (*n* = 34) were morphologically identified as adults of *D. reticulatus*. Twenty-eight ticks were found in all transects from Site A and the remaining six were collected in Site B. Blast analysis of mitochondrial sequences confirmed the morphological identification of processed tick specimens by revealing a highest nucleotide similarity (99–100 %) with those of *D. reticulatus* available in the GenBank database. The phylogenetic trees were concordant in clustering *D. reticulatus* in a monophyletic clade. Seven dogs (8.8 %) had antibodies against *B. canis,* most of which (*n* = 6) came from Site A. Analysis of nucleotide sequences obtained from one tick and from one dog identified *B. canis* displayed a 100 % similarity to those available in GenBank.

**Conclusions:**

This study morphologically and molecularly confirms the presence of *D. reticulatus* in Italy and links it, for the first time, with the occurrence of *B. canis* infection in dogs in this country.

## Background

The tick species most frequently found on dogs in Europe is *Ixodes ricinus* in central and southern Europe, *Rhipicephalus sanguineus* (*sensu lato*) (*s.l*.) in countries on the Mediterranean basin and *Dermacentor reticulatus* in north-central and eastern Europe [1–3]. Further, in eastern Europe the dominance in dogs of *Rhipicephalus rossicus* was demonstrated whereas in northern Europe *Ixodes hexagonus* was one of the most common tick of dogs [4, 5].

The distribution of tick-borne pathogens often overlaps that of their tick vectors; even if the presence of a potential tick vector does not imply the presence of the transmitted pathogen, tick distribution is indicative for the risk of infection to receptive hosts and for setting control strategies against tick-borne diseases (TBDs), at individual and population level [6]. *Dermacentor reticulatus*, the “ornate dog tick” or the “marsh tick”, is a Palaearctic species with an ecologically limited and mosaic distribution pattern mostly associated to cold and wet sites, as well as to host availability [7, 8]. Immature stages are commonly found on small mammals, with a strong preference for the bank vole (*Myodes glareolus*), whereas the adults infest large ungulates, carnivores, horses and wild boars [8–11]. The typical biotope of *D. reticulatus* is represented by forest paths and lakeshore vegetation in association with river basins and swampy mixed woods (i.e. *Querceto populetum, Populetum mixtum, Saliceto-Populetum*) and shrub pasture communities [8]. The geographical distribution of *D. reticulatus* spans from southwestern England to Central Asia and it recently expanding in many countries, such as Germany, Poland, Hungary, Slovakia, Netherlands and Belgium [7, 12–15], but not occurring in the Mediterranean climatic zone [16]. Amongst others, *D. reticulatus* may transmit *Babesia canis*, *B. caballi*, *Theileria equi*, *Francisella tularensis*, *Anaplasma phagocytophilum*, *Coxiella* spp., *Rickettsia* spp. and some tick-borne encephalitis viruses [7]. Canine babesiosis by *B. canis* is primarily focally distributed in central Europe, whereas in the Mediterranean basin it is mainly caused by *Babesia vogeli*, where it is associated with the distribution of *R. sanguineus* (*s.l*.). In northern Italy, a few data support the hypothesis that *B. canis* infection could have an endemic occurrence [17–19]. Following the sole report of *D. reticulatus* in Italy [20], this tick species has never been reported again in this country and *B. canis* was detected only molecularly in a few animals from central and northern Italy [17, 21].

Based on the occurrence of two clustered clinical cases of canine babesiosis in two areas of northeastern Italy (Lombardy region) referred by veterinary practitioners and confirmed by serological tests, a study was planned in order to (i) determine the seroprevalence of babesial infection in dogs; (ii) investigate by molecular tools the etiological agents involved in the cases of canine babesiosis; and (iii) identify the potential tick vector of *Babesia* spp.

## Methods

### Study area

**Fig. 1:**
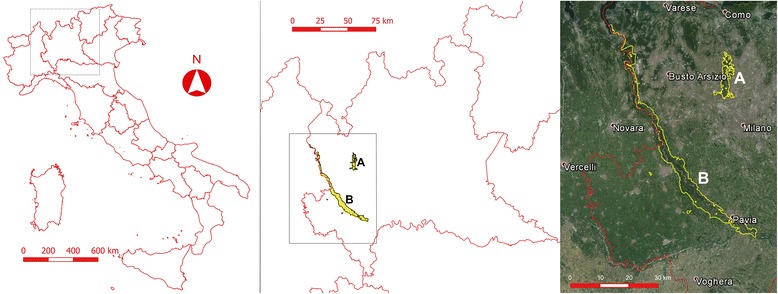
Map of location of the two study areasGroane Regional Park (Site A) (**a**) and the Ticino Valley Lombard Park (Site B) (**b**) in Lombardy (northeastern Italy)

The study area is represented by two parks both located in the eastern part of the Lombardy region (northeastern Italy; 45°34'37.87"N, 9°5'3.96"E) (Fig. 1). Groane Regional Park (Site A) covers 3,400 ha mainly in the northwest of the province of Milan and includes 17 municipalities distributed between two provinces (Milan and Monza Brianza). The environment is characterised by the presence of several rivers and ponds and largely covered by forest with a dominance of *Quercus peduncolata*, *Q. sessiliflora*, *Carpinus betulus, Pinus silvestris* and *Robinia pseudoacacia.* The area is characterised by a sub-oceanic climate, with a mean temperature of 12.4 °C, and an annual rainfall of 1,000 mm (ARPA, http://www.arpalombardia.it/arpa_splash/splash.asp).

The Ticino Valley Lombard Park (Site B) covers 91,000 hectares disseminated along the homonymous river extending between Lake Maggiore and River Po in the western part of Milan province and includes 47 municipalities distributed among three provinces (Milan, Pavia and Varese). Site B presents a wide variety of habitats, including wetlands, watercourses, woods, rice paddies and water meadows. A broad diversity of spontaneous vegetation, due to different ecological conditions is present including tree and shrub species (e.g. *Quercus robur*, *Q. pubescens*, *Q. petraea*, *Carpinus betulus*, *Populus nigra*, *P. alba*, *Salix* sp., *Alnus glutinosa*, *Corylus avellana*, *Acer campestre* and *Crataegus monogyna*). The climate of Site B has a typically continental pattern, with a mean temperature of approximately 13 °C and an annual rainfall of 959 mm (ARPA, http://www.arpalombardia.it/arpa_splash/splash.asp). Both sites are inhabited by a variety of wildlife species, including the red squirrel (*Sciurus vulgaris*), weasel (*Mustela putorius*) and red fox (*Vulpes vulpes*). The roe deer (*Capreolus capreolus*) was recently reintroduced into the Site B. The presence of a range of wild birds and reptiles has also been reported (http://www.parcogroane.it, [22]).

### Tick collection and animal blood sampling procedures

**Fig. 2:**
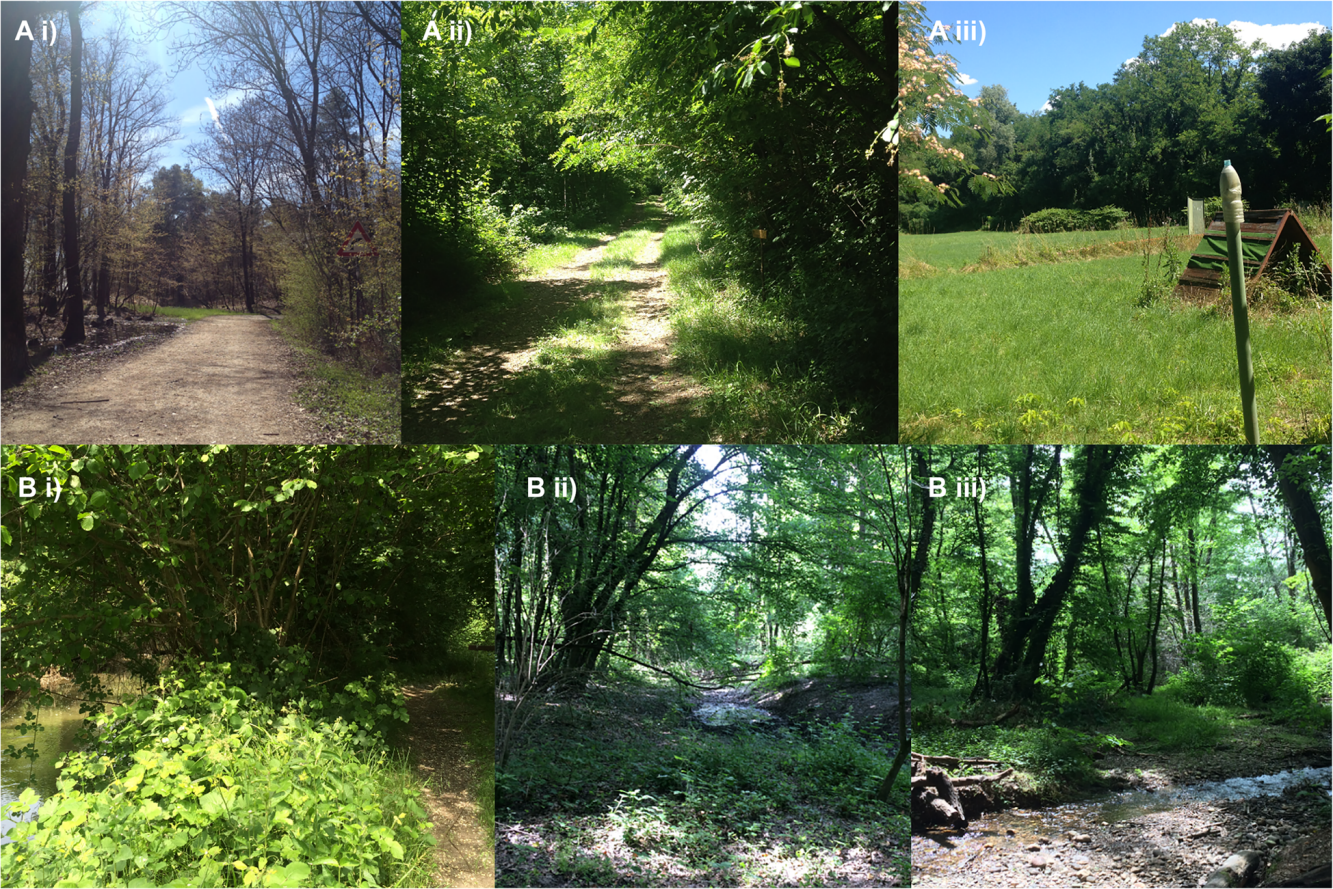
Collection sites of ticks in Groane Regional Park (Site A) and the Ticino Valley Lombard Park (Site B). SiteA (*top*): **Ai**)marked trail, **Aii**) bike trail, **Aiii**) dog training camp; Site B (*bottom*): **Bi**) marked trail, **Bii**) marked trail, **Biii**) isolated wood

From March to May 2015 ticks were collected with a woollen blanket (100 × 90 cm) using both dragging and flagging techniques, on the ground and the bushes in both areas, respectively. Tick collection was performed in three transects in each park, chosen for being compatible with habitat potentially infested by ticks and for the constant presence of pounds or river basins (Fig. 2). In Site A, ticks were specifically collected in a marked trail for pedestrian with picnic site, closer to human inhabited area (45°38'15.5''N, 9°5'31.69"E) (Fig. 2, Ai), a bike trail near a recreational sites (45°37'46.5"N, 9°5'53.7"E) (Fig. 2, Aii) and a dog training camp (45°37'20.9"N, 9°5'34.6"E) (Fig. 2, Aiii). In Site B collections were conducted in a marked trail near a farm (45°19'39.2"N, 8°55'26.3"E) (Fig. 2, Bi), a marked trail near a house (45°20'28.01"N, 8°55'21.9"E) (Fig. 2, Bii) and an isolated wood within agricultural field, next to a country road (45°19'57"N, 8°56'2.7"E) (Fig. 2, Biii). Each sampling session was performed by two operators along a 100 m straight transect. The blanket was inspected every 2 metres, the ticks attached were collected using tweezers and placed in vials containing 70 % ethanol. All sampling sessions started in late morning (11 am) and were carried out during sunny days.

In the same period, blood samples were collected from 80 dogs randomly chosen from veterinary clinics and kennel located in the two studied areas, during routine pre-operatory screenings with the owner's consent. Sample size (i.e. 34.5 animals) was defined according to Daniel [23], based on an expected prevalence of 10 %, a 95 % confidence interval and a precision of 10 %. Therefore, a sample of 40 dogs was established in each study area but the consent was asked to 70 dog owners in each area assuming a high level of refusal. A questionnaire provides data on individual information on life conditions (owned/stray), age (young/adult), life-style (indoor/outdoor), gender (male/female) and breed (pure breed/mixed) of dogs. Further, information of travel history, medical history, antiparasitic treatment and testing for vector-borne infections, was collected. Anamnestic data about enrolled dogs for this study reported no travel history abroad, in European areas endemic for babesiosis, or in other Italian regions. No tick infections were detected at the time of sampling and only two dogs had a precedent exposure to ticks. The whole blood samples obtained from the cephalic vein were stored in sterile tubes and then preserved at +4 °C for a maximum of 3 days or immediately centrifuged at 1500 *g* for 10 min to obtain serum. Serum and whole blood samples were stored at -20 °C until tested.

### Morphological and molecular identification of ticks

Morphological identification of ticks was performed by using taxonomic keys [2, 24, 25]. After morphological identification, six tick specimens (three males and three females) were selected for genetic studies. Genomic DNA extraction was performed using a commercial kit (DNeasy Blood & Tissue Kit, Qiagen GmbH, Hilden, Germany), in accordance with the manufacturer’s instructions. Tick specimens were molecularly characterised by the amplification and sequencing of partial mitochondrial 12S rRNA, 16S rRNA and cytochrome *c* oxidase subunit 1 (*cox*1) genes (∼400, 300 and 600 bp, respectively) as previously described [26]. The percentage of nucleotide variation (Pwc, %) amongst haplotypes, was calculated using the Kimura 2 Parameter substitution model with Gamma Distributed (Γ) rates among sites [27], implemented in the MEGA6 software [28].

For phylogenetic analyses, sequences herein obtained for all genes and those available from the GenBank database for other *Dermacentor* spp. were included. The phylogenetic relationships were inferred by Maximum Likelihood (ML) [27] analysis, conducted using the General Time Reversible (GTR) model using MEGA6 software for both genes [28]. For each gene, homologous sequences for *R. sanguineus* (*s.l*.) (accession numbers: 12S rRNA gene KC243790; 16S rRNA gene KC243837; *cox*1 KC243880) were used as outgroup.

### Serology by IFAT procedure

Dog serum samples were tested with a commercial indirect fluorescent antibody test (IFAT) (MegaScreen®FLUO *Babesia canis*, MegaCor Diagnostic, Horbranz, Austria) to detect the presence of IgG antibodies against *B. canis*, using canine erythrocytes infected with *B. canis* as antigens. Positive and negative controls were always included and were provided by the company. Starting, sera were initially diluted 1:64 in phosphate-buffered saline solution (PBS) (pH = 7.2), applied to slide wells and incubated in a moist chamber for 30 min a 37 °C. The slides were washed and reacted with fluorescein isothiocyanate-conjugated rabbit anti-Dog IgG (Sigma-Aldrich, Milan, Italy), incubated at 37 °C in a humid chamber for 30 min. The slides were mounted with buffered glycerin and covered with a coverslip. Finally, the slides were examined with a microscope (Axioscop 2, Zeiss) under a fluorescent light (HBO50). Those samples that had fluorescence at a diluition ≥ 1:64 were considered as seropositive. Samples resulting seropositive were then diluted to determine the end-point titre (i.e. the last dilution in which is observed positive reaction).

### PCR amplification and DNA sequencing of *Babesia* spp. from ticks and dog whole-blood samples

For the detection *Babesia* spp., genomic DNA was extracted from 100 μl of whole-blood samples using the Archive Pure DNA Blood Kit (5-Prime, GmBh, Hamburg, Germany). Ticks and blood samples were tested by PCR amplification using primers targeting the 18S rRNA gene of *Babesia* spp. (∼410 bp) following previously published methodologies and amplification protocols [29]. A positive control containing genomic *B. canis* DNA and a negative control without DNA were also included. All PCR products were examined on 2 % agarose gels stained with GelRed (VWR International PBI, Milan, Italy) and visualised on a GelLogic 100 gel documentation system (Kodak, New York, USA). The amplicons were purified and sequenced, in both directions using the PCR primers, employing the Taq Dye Deoxy Terminator Cycle Sequencing Kit (v.2, Applied Biosystems, Monza MB, Italy) on an automated sequencer (ABI-PRISM 377). Sequences were aligned using ClustalW programme [30] and compared with those available in the GenBank database (BLAST,http://blast.ncbi.nlm.nih.gov/Blast.cgi).

## Results

All ticks collected in the surveyed areas (*n* = 34) were morphologically identified as adults of *D. reticulatus*. Briefly, adult specimens of *D. reticulatus* were differentiated by the presence of a spur on the dorsal surface, extending posteriorly, of the second palp articles, a bifid coxa, with coxal spurs in coxa I not divergent, spiracle plates comma-shaped with a small dorsal apofisa. In males, cornua (external margins of the posterior dorsal surface of basis capituli) were prominent and rounded apically, scutum had broad lateral grooves with several small punctations, trochanter 1 bearing a long dorsal posterior spur. Female *D. reticulatus* had oval shaped porose areas on the dorsal surface of the basis capituli, the ornate scutum had sides rather rounded and not particularly angular, the genital aperture lacked wing-like outgrowths and formed a broad U shape, truncated posteriorly, anal groove surrounding anus posteriorly and continued in a posterior post anal groove.

**Fig. 3:**
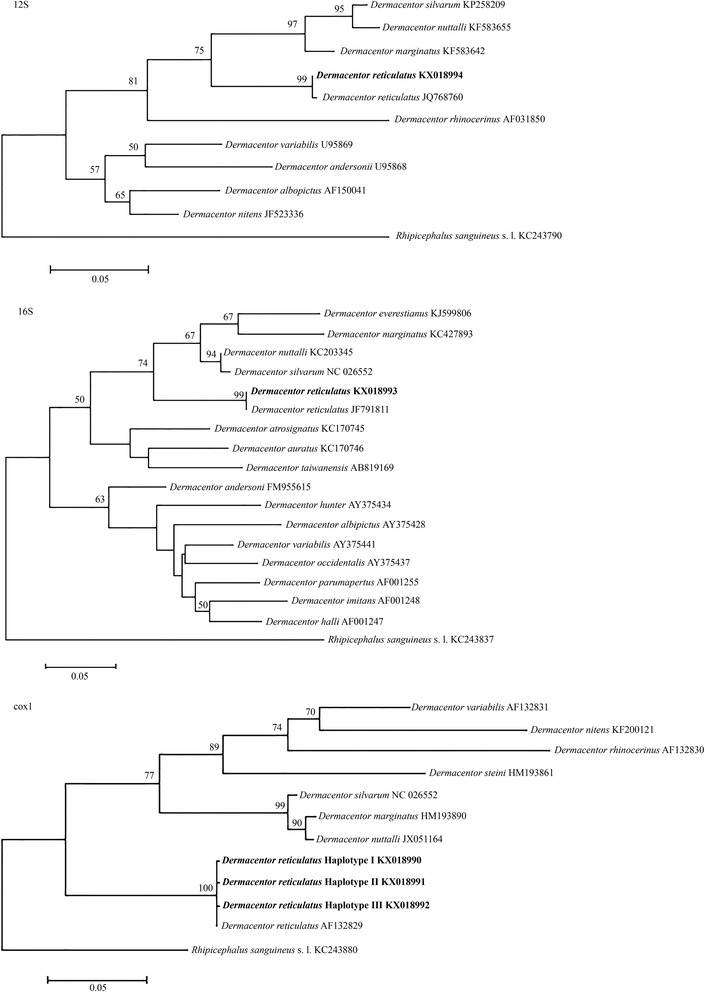
Phylogenetic trees for *Dermacentor* spp. including the newly-sequenced isolates of *Dermacentor reticulatus* (*bold*) and sequences from other species available in the GenBank database. Nodal support bootstrap values > 50 % are indicated only. *Rhipicephalus sanguineus* (*sensu lato*) was used as the outgroup

Of the 34 adult *D. reticulatus* collected from the ground of both surveyed areas, 28 (13 males and 15 females) were found in all transects from Site A and the remaining six specimens (three males and three females) were collected in Site B in two out of three settled transects. Ticks were collected in May (*n* = 19; 56 %), April (*n* = 9; 26 %) and March (*n* = 6; 18 %). Blast analysis of all mitochondrial sequences confirmed the morphological identification of the tick specimens processed by revealing a highest sequence nucleotide similarity (99–100 %) with those of *D. reticulatus* available from GenBank (i.e. 12S rRNA gene: JQ768760; 16S rRNA gene: JF928523; and *cox*1: AF132829). The obtained sequences were deposited in GenBank (accession numbers: KX018990-KX018994). For *cox*1, three haplotypes were identified, with a nucleotide variation of up to 0.4 %. The conceptual translation at second codon position of *cox*1 haplotypes resulted in amino acid sequences with no stop codons. The vast majority of nucleotide substitutions were synonymous substitutions (dS), with the exception of one (second codon position; non-synonymous substitutions dN), which resulted in amino acid alterations (Val-Met). Reflecting the molecular identification, the phylogenetic trees of haplotypes for all genes herein examined and those for other *Dermacentor* spp. retrieved from GenBank, were concordant in clustering *D. reticulatus* in a monophyletic clade with the exclusion of other ticks species (Fig. 3).

A total of seven dogs (8.8 %) had antibodies against *B. canis*, most of which came from Site A (Table 1). Out of the seven dogs, five (6.3 %) and two (2.5 %) were positive at high titres of 1:1.024 and 1:512, respectively. The highest seroprevalence values were observed in May (*n* = 4; 57 %) and April (*n* = 2; 29 %). Information on positive dogs is summarised in Table 1; all of them were owned, none of them had been travelling 12 months prior to sample collection and they had never been in endemic region for *B. canis* infection. Further, none of the sampled dogs had a vaccination for *Babesia. Babesia* spp. DNA was amplified from four out of six ticks processed molecularly, and from three (i.e. two from Site B and one from Site A) out of 80 tested canine whole blood samples (Table 2). Analysis of nucleotide sequences obtained from ticks and from one dog identified with *B. canis* displayed 100 % similarity to that available in GenBank (DQ174284.1).

**Table 1:** Summarised details of ten dogs resulted positive for *Babesia canis* at IFAT or PCR

Dog-ID	Sex^a^	Age (months)	Breed	Clinical signs	Site^b^	PCR	IFAT
1	M	120	Mixed	none	A	Negative	1/1024
2	M	108	Mixed	hemoglobinuria	A	Negative	1/1024
3	M	60	Mixed	none	A	Negative	1/1024
4	M	48	Mixed	none	A	Negative	1/512
5	F	72	Mixed	none	A	Negative	1/1024
6	F	12	Mixed	none	B	Positive	Negative
7	F	108	Italian griffon	none	B	Positive	Negative
8	F	12	Mixed	none	B	Negative	1/512
9	M	72	Chow chow	weakness fever	A	Positive	Negative
10	M	84	Hovawart	None	A	Negative	1/1024

^a^Sex: F, female; M, male

^b^Site A: Groane Regional Park; Site B: Ticino Valley Lombard Park; IFAT: serum titre

**Table 2:** Results of *Babesia canis* tests on dog samples and ticks from two surveyed areas in northeastern Italy

Samples	Site A				Site B				Both sites
	IFAT	PCR		Total	IFAT	PCR		Total	
		Positive	*B. canis* confirmatory sequencing			Positive	*B. canis* confirmatory sequencing		
	N/tested^a^ (%)	N/tested^a^ (%)	N/tested^a^ (%)	N/tested^a^ (%)	N/tested^a^ (%)	N/tested^a^ (%)	N/tested^a^ (%)	N/tested^a^ (%)	N/tested^a^ (%)
Dogs^b^	6/57 (10.5)	1/57 (1.7)	1/1 (100)	7/57 (12.3)	1/23 (4.3)	2/23 (8.7)	–	3/23 (13.1)	10/80 (8.0)
Ticks	–	3/3 (100)	1/3 (33.0)	3/3 (100)	–	1/3 (33.0)	–	1/3 (33.0)	4/6 (66.7)

^a^N/tested = number of positive samples/number of tested samples

^b^Sera or whole-blood samples

## Discussion

This study morphologically and molecularly confirms previous sporadic reports of *D. reticulatus* in Italy [20, 31] and links, for the first time, its presence with the occurrence of *B. canis* infection in dogs in this country. Our data represent one of the southernmost findings of *D. reticulatus* in Europe, which is usually reported in Switzerland [32], France [33, 34], Austria [35], Croatia [36] and Slovenia [37]. In addition, since a few specimens of *D. reticulatus* have been so far collected below the limit of 50°N (e.g. in Portugal, Romania and Hungary [7]) data here presented confirm that this tick species may occur even below the 45°N perpetuating its biological life-cycle in regions of the Mediterranean basin, where meteorological conditions compatible with tick requirements occur (e.g. spring precipitation of 400–1000 mm and a summer isotherm of 20–22 °C) [38].

The environment of both parks, in the area of low plain of Po Valley, is suitable for the life-cycle of *D. reticulatus*, since they are characterised by clay, loam and rich of waters of different origins, consistent with the typical biotope of this tick species [7, 8]. Moreover, by the end of 1800's through the 1950's, at the time when this territory was mostly characterised by swampy lands, paddies and water-meadows continuously irrigate to allow the growth of grass also in the cold winter, this tick species might have been even more widespread compared to the present. In the past decades, the extensive changes of the territory, as well as the strong reduction of water-meadows replaced by intensive agricultural practices mainly aimed to produce cereals and the increase of urbanisation occurred in that area may have reduced the suitable areas for *D. reticulatus* that are waste lands [7, 39]. Moreover, the wetland environments of the surveyed parks are preserved by the European rules (Dir. 79/409/CEE) and both parks are rich of animal species that could act as potential suitable hosts (e.g. dogs, horses, cattle, wild ungulates and the bank vole). *Dermacentor reticulatus* were collected in rural areas close to urban settings hence confirming previous findings of this tick species proliferating in waste lands situated between the blocks of apartments of large residential estates where it was supported by the increase of canine population in the areas [39] and even in towns [7], emphasising the sanitary risks for human beings [6].

*Dermacentor reticulatus* is exo-endophilic (adult ticks occur on the vegetation whereas immature ticks are associated with nests), ditropic (immature and adult ticks feed on different host species), and three-host (each life-cycle stage requires a new host to feed on) tick species, being the adult stages usually associated with large mammals (horses, cattle, dogs and wild ungulates) and the juvenile stages with several species of micro-mammals [2, 40]. In the surveyed areas this tick species was most likely associated with dogs (horses and cattle are confined in stables and in farms) though wild ungulates, such as the roe deer (*Capreolus capreolus*), transit in the Ticino Valley Lombard Park, which is known as an ecological corridor for this wild species. In addition, in both collection sites the bank vole (*Myodes glareolus*), which is the favourite host for the immature stage of *D. reticulatus*, is highly prevalent [9].

The number of ticks collected in the surveyed areas is consistent with the findings of other surveys carried out in neighboring countries with similar ecological and host features, but is lower than that of *D. reticulatus* in the western and eastern Europe [32].

The seroprevalence of *B. canis* here detected (8.8 %), also confirmed by molecular tests and gene sequencing of the pathogen, was lower than that previously reported in northeastern, central and southern Italy (i.e. 70 %; see [21, 41]). Such, a prevalence of infection of *B. canis* in southern Italy most likely indicates the failure of serological tests in differentiating anti-*B. canis* and *B. vogeli* specific antibodies [42, 43]. Although in previous studies the occurrence of ticks on hosts has never been investigated thoroughly [21, 41], it is known that *R. sanguineus* (*s.l*.), the vector of *B. vogeli*, is the most frequently retrieved tick species but not *D. reticulatus* [20, 44].

Whether *B. canis* has been recently introduced in the studied area or it is present since along time is difficult to be assessed. Nonetheless, the studied area is historically known as endemic for canine babesiosis. Indeed, Piana & Galli-Valerio [45] firstly described *Babesia* sp. infection in a hunting dog in the first week of April from an adjacent area of Ticino Valley Lombard Park. The low seroprevalence actually recorded in the studied area may also be the consequence of a reduction of tick infestations on dogs due to the increase of awareness and education of owner and veterinary practitioners, along with the increase of use of acaricide products among the prophylactic measures in dogs [46, 47].

The IFAT positivity here detected was uncorrelated with PCR results due to the fact that after the decrease of parasitemia, antibodies are still present [21]. However, the molecular detection of *B. canis* in natural infected dogs and, for the first time in Italy, in questing *D. reticulatus* confirms the focal nature of the infection. Recently, *B. canis* was molecularly characterised in two out of three *Dermacentor marginatus* (but not *D. reticulatus*), morphologically identified, from dogs in northern Italy [48], hence raising some doubts on the morphological characterisation of those tick species that is associated to wild and domestic ungulates, and only seldom to dogs and humans [2, 24, 25]. Considering also that *D. marginatus* should not be the competent vector for *B. canis* being that to the best of our knowledge, no data are available to simultaneously demonstrate the molecular identification of both *D. marginatus* and *B. canis*, then *D. reticulatus* and consequently *B. canis* may occur in other areas of Italy, outside the surveyed foci.

Finally, the nucleotide identity of *B. canis* from dogs and ticks indicates that the same strain circulates in the studied area among vectors and definitive hosts. The endemic nature of canine babesiosis by *B. canis* has been previously described in Germany [49], Croatia [50], Slovenia [51], Hungary [52], Russia [53], Spain [54], Portugal [55], Albania [56], Slovakia [57], Serbia [58], Switzerland [32], Netherlands and Belgium [14], Poland [59] and France [34].

The occurrence of *D. reticulatus* in sites from two parks of northeastern Italy located in a highly urbanised area indicates that risk factors linked to *B. canis* in dogs are not confined to hunting dogs as in the past [60], but infections also reaches owned dogs living in small urban centres included in or close to natural environments.

## Conclusion

This study demonstrates that *B. canis* and its vector, *D. reticulatus* ticks, circulate in northeastern Italy. Although *D. reticulatus* only seldom bites humans, its capacity as a vector of zoonotic pathogens (e.g. *Rickettsia slovaca*, *R raoultii* and *R. helvetica*) sounds as an alarm bell for physicians working in areas where this tick species may occur.
